# Defining Spotting in Dance: A Delphi Method Study Evaluating Expert Opinions

**DOI:** 10.3389/fpsyg.2021.540396

**Published:** 2021-05-13

**Authors:** Catherine Haber, Andrea Schärli

**Affiliations:** Institute of Sport Science, Dance Science, University of Bern, Bern, Switzerland

**Keywords:** rotation, head coordination, orientation, timing, ballet, pirouette

## Abstract

Spotting is a typical isolated head coordination used by many dancers during rotation. However, with sporadic and inconclusive explanations as to why dancers spot, the critical characteristics and functionalities of spotting have yet to be identified. Therefore, a Delphi method survey was used as a novel methodology for providing greater insights into this under-examined motor behavior, bringing together experts from various disciplines to generate ideas and identify the crucial elements of spotting. Following the selection of experts, three rounds of data collection and analysis were conducted to narrow down relevant topics and evaluate consensus. To gather opinions in Round 1, experts were asked to respond freely to three prompts regarding the reasoning, characteristics, and uses of successfully spotting; responses were then grouped into predominant items. To rate agreement in Round 2, experts rated their agreement on the relevance of the grouped items from Round 1 on a 5-point Likert scale; items rated 4 or 5 by at least 70% of the experts were taken as those consensually relevant to the group. To rank importance in Round 3, Best-Worst Scaling was used to determine individual rankings of the relevant items from Round 2. In a series of comparisons, experts were prompted to select the most and least important items in multiple sub-groupings. Group mean ranking of items as well as ranking concordance and differentiation were analyzed to determine the most important items and the strength of consensus, respectively. Overall, consensus and differentiation in experts’ item rankings were low; however, novel insights were presented. As characteristics of successfully spotting, experts emphasized head isolation, timing, and gaze specificity alongside functional characteristics, substantiating spotting as purposeful action in rotation. Building on traditional notions of spotting for reduced dizziness and maintaining balance, successfully spotting was further deemed useful for multiple turns, orientation, and rhythm. The findings of this study thus provide informed guidelines for future analysis and training of this complex head coordination in rotations.

## Introduction

Preparing to turn, a dancer fixes the head and gaze toward a visual object at eye level as the body begins to rotate. When the head reaches maximal rotation and the fixation can no longer be sustained, the head quickly rotates, overtaking the body to return to the same spot. This technique is known as spotting. While spotting is an integral element to rotation in dance styles, such as classical ballet and modern dance, similar rotations in gymnastics or ice-skating lack such an isolated head coordination. So why do dancers spot? With the technique so central to practice – particularly in ballet – several explanations have been put forward from historical and physiological perspectives as well as from limited biomechanical research. However, to date, no consensus on the relative merit of each of these explanations has been achieved.

From a historical perspective, the development of classical ballet has been influenced by the potentially irreversible accumulation of techniques – beneficial or not – that have been carried through generations (Hammond and Hammond, 1999). While dance technique manuals of the 18th and 19th centuries made no mention of spotting, Hammond and Hammond (1999) claim that spotting had undoubtedly long been in use. Spotting only gained attention in the 19th century when multiple revolutions became popular in ballet. In 1893, Pierina Legnani’s performance of 32 fouettés (i.e., multiple rotations on one supporting leg while continually swinging the gesture leg) drastically increased the number of revolutions thought possible. Many dancers accordingly adopted her technique of rapid spotting. One of the earliest written mentions of spotting appeared in the memoir of Mathilde Kschessinska, the first Russian ballerina to accomplish Leganani’s feat, stating: “To dance fouettés in one spot one has to have a clearly visible mark ahead every time one turns” (Kschessinka, 1997, p. 76). Given the general style of classical ballet to perform toward the audience, this forward-looking head motion was additionally suggested to support the aesthetic of ballet, particularly in fouettés, where the dancer sustains the head orientation toward the audience for a significant portion of each revolution (Laws, 1986).

Following explanations of spotting’s utility to stay in place or for aesthetical appearances, spotting was considered an integral technique for maintaining balance during and when stopping rotation (Tschiassny, 1958; Osterhammel et al., 1968). Such claims were generally accompanied by physiological explanations, framing spotting as a mechanism to reduce vestibular stimulation. Information from the vestibular system is especially important to dancers as it informs balance and orientation. Housed within the inner ear, the vestibular system provides sensory feedback on angular and linear accelerations in response to displacements of hairs and fluids within its structure (Obrist, 2011). Specifically, motion causes changes in the inertia of the endolymph that flows through the semicircular canals. This change in the endolymph deflects the cupula, activating sensory cells that signal the sensation of motion. With regard to ampullary cupula displacement during rotation, Tschiassny (1958) hypothesized that the quick acceleration and retardation of the head during spotting neutralize one another when occurring in such a short interval, therefore decreasing vestibular receptor displacement and post-rotary reactions.

Therefore, several empirical studies observed dancers on rotating chairs with electrooculography measures of post-rotary nystagmus in the horizontal eye plane. During and after continued rotation, the eyes make involuntary, repetitive movements known as nystagmus. With this physiological response, the body aims to correct for disturbances in the visual field that occur during rotation. As the intensity of the nystagmus directly corresponds to the magnitude of cupula displacement, vestibular stimulation during and after rotation can thus be measured (Obrist, 2011). In general, dancers were found to have a significant degree of habituation to rotation – independent of the spotting technique – as marked by lower vestibulo-ocular responses and lower subjective feelings of vertigo (Tschiassny, 1958; Osterhammel et al., 1968; Teramoto et al., 1994; Nigmatullina et al., 2015). Specifically, Teramoto et al. (1994) observed a relationship between years of ballet experience and an individual’s level of post-rotatory nystagmus. Ballet dancers with more years of experience exhibited lower levels than less experienced dancers, whereas dancers with less than three years of experience exhibited responses similar to those of non-dancers. Even when displaying strong levels of nystagmus similar to that of control participants, experienced dancers subjectively reported little to no dizziness (Osterhammel et al., 1968). An even further reduction of post-rotary responses was observed when dancers employed the spotting technique on the rotating chair, again, with greatest reductions observed in more experienced dancers (Tschiassny, 1958; Osterhammel et al., 1968).

Countering the findings in the dancer population, a more recent study examining the influence of visual fixation during and after rotation in non-dancers found no benefit of spotting (Kim et al., 2013). Participants were rotated (1) without visual fixation, (2) fixating while rotating with the spotting technique, (3) fixating while rotating (spotting) and fixating after rotating, and (4) fixating only after rotation. No improvements were found from spotting alone (2), but only when participants fixated after rotation (3 and 4 providing similar results). In this cohort, spotting was found to have no influence on subjective dizziness nor vestibulo-ocular responses. The authors support this finding with the following explanation. Since post-rotary dizziness is caused by the oppositional inertial flow of endolymph when the rotation stops, fixation during rotation would not influence the inertial flow afterward. Rather, fixation afterward inhibits post-rotary nystagmus, thus inhibiting post-rotary dizziness.

While opposing findings have been presented for spotting in passive rotations, Tokita et al. (1972) examined rotary nystagmus in the performance of active ballet rotations. The irregular per-rotary nystagmus (i.e., nystagmus during rotations) observed in novice dancers appeared to be more controlled in experienced dancers. These experienced dancers rather displayed a one-beat nystagmus per rotation in the turns of tour chaînés (i.e., traveling turns), pirouettes (i.e., a balanced 360° turn on one leg), and fouettés. The authors concluded that these intentionally controlled eye movements were coordinated with the vestibulo-ocular reflexes of the jerking head movements, thus training control over per-rotary nystagmus. Similarly considering effects of expertise in active rotation, Hopper et al. (2014) found that while recreational and pre-professional dancers increased postural sway after performing multiple rotations, professional dancers’ balance was unaffected. Again, the authors suggest the importance of spotting for balance and cite the findings of McMillan (1972) that expert dancers perform a different spotting technique than novices.

Taken together, the study of spotting in passive and active rotations suggests the greatest benefit with greater experience and training in the technique; however, the characteristics of performing an optimal spotting technique remain unclear. From a biomechanical perspective, spotting is not functional for rotation. In the preparation of a turn, head movement has been found to have negligible effects on the generation of angular momentum to initiate rotation in a pirouette (Kim et al., 2014). During a turn, spotting in fact has a negative effect on the rotational speed. The biomechanical analysis of turns by Laws (1978) illustrates that the whipping movement of the head actually absorbs some angular momentum from the turn, and as a result slows the body’s rate of rotation. Therefore, to reduce this slowing effect of spotting, the dancer must keep the head on the axis of rotation where it has a smaller moment of inertia.

In terms of the spotting technique itself, two studies have examined the head coordination during rotation. The early cinematographic work of McMillan (1972) identified that skilled dancers return the head to the spotting point earlier in rotation than non-skilled dancers. Later on, Lin et al. (2014) examined the head coordination throughout a turn. Here, the Anchoring Index (AI), a measure generally used to indicate segment coordination in walking stability, was applied to analyze the head-on-trunk coordination during single pirouettes. Expert dancers exhibited more positive AI’s, indicating the stabilization of the head with reference to the global axis, whereas novices had more negative AI’s, indicating the use of a local reference or an en bloc strategy with the torso. Thus, the authors attribute greater head stabilization to expert performance and suggest the role of this stabilization in processing visual inputs. Regarding visual information, the height of a spotting fixation point has been found to influence preparation and landing dynamics in single pirouettes (Cicchella and Caminiti, 2015). Specifically, a very low fixation point increased the preparation duration and time to peak force on the supporting leg; however, head coordination was not directly examined in response to varying visual stimuli.

In summary, the reviewed literature proposes that spotting is useful for: staying in place (Kschessinka, 1997), aesthetically pleasing audiences (Laws, 1986), reducing subjective dizziness, and objectively measured vestibulo-ocular responses in dancer populations (Tschiassny, 1958; Osterhammel et al., 1968; Teramoto et al., 1994; Nigmatullina et al., 2015), or maintaining balance (Tschiassny, 1958; Osterhammel et al., 1968). The spotting technique itself has been shown to have no effects on non-dancer populations (Kim et al., 2013) and negative effects on whole body turn rate (Laws, 1978). Yet, somehow dancers are able to train the controlled reflexes of spotting for improved post-rotary responses. From arguments that spotting is under visual control, the findings of Cicchella and Caminiti (2015) support that dancers are affected by changes in fixation height and, therefore, may be dependent on visual input. On the other hand, perhaps it is the space-referenced head coordination of experienced dancers, reducing vestibular stimulation, that is most important (Lin et al., 2014). While providing sporadic insights, the research to date lacks a clear understanding of spotting. Thus, the questions remain: Why do dancers spot? What characterizes successfully spotting? What is successfully spotting useful for?

As an intermediary step toward answering such questions, one may call on the insights of experts in the field. The input of expert knowledge in empirical settings has been claimed to be increasingly advantageous, particularly when it comes to the identification and description of behavioral acts (Kirker et al., 2000). Such valuable contributions elucidate the language and perspectives used to best describe phenomena of interest. Given the limited research on spotting, the input of experts can: highlight relevant aspects of practice, provide hypotheses for more directed movement-based research, and ensure that results of such empirical work transfer to applicable elements in the field. Therefore, in order to first bring together the ideas of an expert panel, a Delphi method survey is a valuable tool to elicit expert opinions over iterative rounds with controlled feedback. Thus, the aim of the present study is to answer the three questions posed above regarding the reasoning, characteristics, and functionality of spotting with a Delphi method survey, gathering expert inputs to build and evaluate consensus on spotting.

## Materials and Methods

**FIGURE 1:**
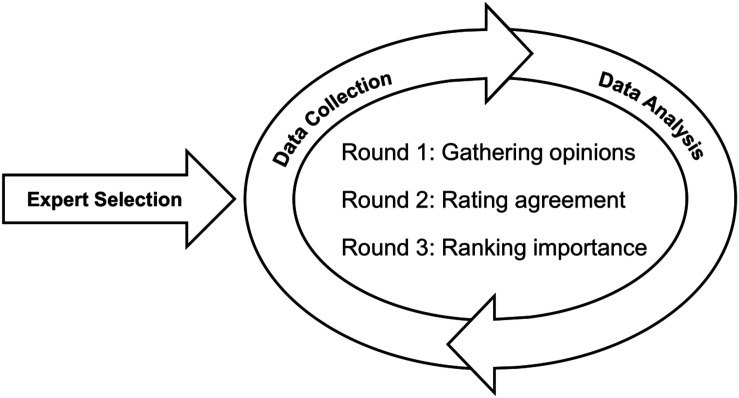
Schematic representation of the three-round, ranking-type Delphi methodology.

The Delphi methodology used in the present study is adapted from the guidelines of Kobus and Westner (2016) for ranking-type Delphi studies. After identifying expert categories and selecting experts, three rounds of integrated data collection and analyses were performed. In each round, once expert inputs were collected, the responses were analyzed to inform the development of the survey for the following round. To this end, the first round aimed to gather opinions, the second round to rate agreement on relevant items put forth in Round 1, and the third round to rank the importance of the relevant items from Round 2. Accordingly, the iterative and cyclical process of the Delphi methodology is depicted in Figure 1.

### Expert Selection

As the Delphi method is highly dependent on the group of experts contributing, experts were defined and selected carefully. Hsu and Sandford (2007) recommend that the experts selected should have related experiences and backgrounds in the topic, be able to contribute meaningful insights, and/or be trained in or competent with the specified area of knowledge. To address the topic of spotting, it was determined that experts from the field of ballet should be selected, as spotting is an explicit component of this dance style (Laws, 1978; Hammond and Hammond, 1999). In this regard, ballet teachers – who teach the spotting technique – and professional ballet dancers who masterfully use the spotting technique – were identified as two important expert categories that have practical experience with spotting. Additionally, dance scientists were identified as the third expert category, adding experts in the study and analysis of dance and movement. With these three categories, a wide breadth of knowledge on spotting would be represented, preventing a distortion of findings based on the perspectives of a singular group (Paré et al., 2013). Moreover, given the sporadic scientific research on spotting, integrating the voice of practitioners (i.e., ballet teachers and professional ballet dancers) would allow for the support or rejection of empirical hypotheses proposed by dance scientists or, further, for the introduction of novel aspects that have yet to be empirically examined. The selection criteria required that experts hold one or more of the following qualifications:

(1) Ballet teachers: Individuals teaching (pre-)professional ballet on a regular basis for at least 5 years.
(2) Professional ballet dancers: Individuals performing with a professional ballet company for at least 5 years.
(3) Dance scientists: Individuals holding a higher education degree in Dance Science or Human Movement Science.

The optimal panel size was determined to be 30 experts, since groups of heterogeneous expertise tend to be bigger and groups larger than 30 rarely generate novel ideas or improve results (Villiers et al., 2005; Hsu and Sandford, 2007). Following expert category selection, expert contacts were identified. An initial list of 27 experts was generated, utilizing the authors’ personal networks as a reliable resource for recruiting specialists (Paré et al., 2013). Experts from multiple institutions across North America, Europe, and Australia were invited *via* email to participate in the study. The experts who agreed to participate were then asked to nominate additional experts, resulting in the final group of 30 expert participants.

### Data Collection and Analysis

Given that the Delphi method is iterative and sequential, the integrated data collection and analysis for each round will be presented together. Three rounds were employed, as three rounds are generally sufficient and more commonly used (Hsu and Sandford, 2007; Diamond et al., 2014). Three online surveys were designed to, respectively, gather opinions (Round 1), rate agreement (Round 2), and rank importance (Round 3) of the reasons, characteristics, and uses of spotting. At the end of each survey, participants were given space to contribute additional comments.

All rounds took place within a 2.5-month period. After the initial Round 1 invitation, Round 2 and Round 3 surveys were distributed 23 and 47 days after their preceding rounds, respectively. Maintaining participant engagement, the timing of the distribution was faster than or in line with the average Delphi duration of 45 days between rounds (Villiers et al., 2005). Participants were given 2 weeks to respond to each survey. Those who did not respond to a prior round were not invited to participate in the following round (Murry, 1995).

#### Round 1: Gathering Opinions

The aim of Round 1 was to discover the relevant issues through a form of brainstorming (Schmidt et al., 2001; Kobus and Westner, 2016). Therefore, experts were prompted with open-ended questions to elicit their opinions on the topic (Murry, 1995). The first-round survey was distributed electronically via Google Forms (Google Inc.). Experts first provided informed consent and demographic information, including age, gender, identification with the three expert categories noted above, years of experience in each of the three expert categories, and for those who indicated teaching experience, the age groups and skill levels they taught. Experts were then asked to “Describe spotting in [their] own words” to bring to mind the topic at hand. Followingly, experts were presented with the three main prompts regarding the reasons, characteristics, and uses of spotting:

(P1) Why do dancers spot?
(P2) Successfully spotting is characterized by:
(P3) Successfully spotting is useful for:

The first prompt (Why do dancers spot?) posed a neutral starting question that invited experts to bring to mind their first reactions on the topic before calling on more specified or analytical characteristics. As spotting is performed within the embodied artform of dance, the authors remained open to the possibility that the reason for performance could be stylistic rather than functional. Nevertheless, stylistic requirements can additionally provide functionality (e.g., external rotation of the hips, or turnout, in ballet stems from historical origins, though it is useful for increasing the range of motion in leg extensions). Therefore, the third prompt (Successfully spotting is useful for:) aimed to specifically identify the uses of the coordination – regardless of whether the reasons for performance were functional or not. To avoid the subjectivity of a good or bad spotting technique, “successfully spotting” aimed to focus on the key elements of spotting performance. Experts were invited to respond freely to the prompts above either in a list or in full descriptive text as they felt fitting.

The first-round analysis encompassed the consolidation and grouping of the responses from Round 1 into items to be used in Round 2. Stemming from the principles of content analysis, the criterion for selection and levels of abstraction were first determined for the inductive development of items, aiming as close as possible to the presented material (Mayring, 2000). To address the particularity of movement description, consolidation of items intended to hold the experts’ wording as closely as possible and to avoid any reinterpretation of the researchers. For this reason, items were created and grouped based on the exact wording. Specifically, the first author initially reviewed all responses from the open-ended questions in Round 1. Responses that were not already in list form were reformatted as such by identifying key quotations. Quotes that used the same wording (e.g., “dizziness”) were then grouped, such that all answers with unique wording were represented. At this stage, the second author reviewed all quotes and proposed groupings, which were then finalized by the two authors together. The final items were subsequently created based on the predominant words within each grouping and phrased for consistency within each prompt [e.g., for P1 (Why do dancers spot?), all items started with “To…”].

#### Round 2: Rating Agreement

The aim of Round 2 was to narrow down the most relevant items from Round 1 (Schmidt et al., 2001; Kobus and Westner, 2016). In order to determine which items the expert group agreed to be most relevant, a rating strategy was used in another Google Forms survey (Villiers et al., 2005; Hsu and Sandford, 2007). The experts were once again presented with the three prompts and the respective summarized items of each prompt. As in the one-round, dance-specific Delphi study of Lerch et al. (2017), experts were instructed to rate their agreement with each item in relation to its respective prompt on a 5-point Likert scale. For the analysis of Round 2, items that were rated 4 or 5 (agree or strongly agree) by at least 70% of the group were taken as those consensually agreed to be relevant (Villiers et al., 2005).

#### Round 3: Ranking Importance

A key element of the ranking-type Delphi study, the third and final round entailed ranking the importance of the relevant items from Round 2 (Schmidt et al., 2001; Kobus and Westner, 2016). In order to determine an individual’s strength of preference for each item, a Best-Worst Scaling (BWS) method was used. BWS is a discrete choice technique that determines individual rankings based on a series of choices (Louviere et al., 2013). Rather than simply numbering items in order of preference, rankings are computed by considering how often an individual chooses one item over the other; specifically, by choosing the best and worst items in a subgrouping of items, or a choice-set. BWS is an advantageous ranking method as it overcomes prominent response biases (i.e., acquiescence bias, extreme response bias), removes idiosyncrasies in response to rating scales (i.e., gender and cultural differences), and provides a clearer discrimination of the relative preference between items (i.e., reliability of “middle” item rankings; Lee et al., 2008).

To this end, the online survey software QuestionPro (QuestionPro Inc.) was used to create and analyze the BWS (or as known in QuestionPro, Maximum Difference Scaling) survey. To ensure that each item is presented equally often and as frequently with every other item, a Balanced Incomplete Block Design (BIBD) was employed in QuestionPro to form the choice-sets (Lee et al., 2008). Based on the number of items resulting from Round 2, the optimal number of choice-sets and items in each choice-set could thus be determined. For each of the three prompts in Round 3, participants were presented with the determined number of choice-sets in a randomized order. Each choice-set displayed varying subgroupings of the relevant items from Round 2. In each case, participants were instructed to select the “Most Important” and “Least Important” items.

As a first step in the analysis of Round 3, individual’s rankings were determined based on the BWS responses. BWS analysis is founded in random utility theory, which assumes that the preference for item A over B is a function of the frequency with which item A is chosen over B and additionally recognizes that individuals can make stochastic decisions with errors in choices (Louviere et al., 2013). Yet, with choice analysis based on the conditional logit model, these errors are assumed to be independently and identically distributed. Therefore, from raw utility values, the share of preference can be calculated for each item. These values were output from QuestionPro and sorted to determine each individual’s rankings. Items with the same share of preference were given an average ranking (e.g., two items with the same highest share of preference each received a ranking of 1.5).

Next, group mean ranking of items and the coefficient of concordance (Kendall’s W) were calculated in SPSS Statistics 24 (IBM) to determine the most important items and the strength of consensus in the group, respectively (Kobus and Westner, 2016). The significance of the differentiation between mean rankings was then determined by a Friedman’s Test with an alpha level of 0.05.

To assess whether the opinions of the whole group were similarly reflected in the opinions of those with similar experiences, *post-hoc* subgroup analysis was performed. The remaining experts in Round 3 were split into two subgroups: dance scientists (i.e., individuals holding a higher education degree in Dance Science or Human Movement Science) and practitioners (i.e., both ballet teachers and professional ballet dancers without dance-scientific experience and solely practical expertise). All measures that were analyzed for the whole group were additionally calculated for these two subgroups. Items in the top half of the subgroup mean rankings were also identified. Finally, additional expert contributions throughout the three-rounds, such as items that were not agreed to be relevant from Round 2 as well as expert comments, were reviewed once again.

## Results

### Expert Selection

The response rate from the initial call to participation was 66% (18 of 27 experts). With the addition of the 12 experts nominated by their peers, the optimal initial group size of 30 experts was obtained. Regarding the expert categories represented (i.e., dance scientist, ballet teacher, or ballet dancer), 11 experts identified with 1 of the 3 expert categories, 13 identified with 2 categories, and 6 identified with all 3 categories. The initial expert group was highly experienced within each category, with 10.8 ± 6.2 years of experience as dance scientists, 18.8 ± 10.5 years as ballet teachers, and 16.7 ± 8.9 years as professional ballet dancers. The first round resulted in a 100% response rate (*n* = 30; age = 45 ± 10 years; and 67% female), the second round in a 90% response rate (*n* = 27; age = 46 ± 10 years; and 63% female), and the third round in an 85% response rate (*n* = 23; age = 46 ± 10 years; and 65% female). Altogether, 77% of the initial 30 experts completed all rounds. While seven participants dropped out over the survey process, the demographic distribution and distribution of expertise remained relatively similar throughout all rounds (Table 1).

**TABLE 1:** Representation of each expert category and years of experience.

	Dance scientist	Ballet teacher	Ballet dancer
**Round 1 (*N* = 30)**			
*n* (% representation)	15 (50%)	21 (70%)	19 (63%)
Mean years of experience (SD)	10.8 (6.2)	18.8 (10.5)	16.7 (8.9)
**Round 2 (*N* = 27)**			
*n* (% representation)	13 (48%)	19 (70%)	17 (63%)
Mean years of experience (SD)	10.7 (6.2)	19.5 (10.3)	17.7 (8.9)
**Round 3 (*N* = 23)**			
*n* (% representation)	12 (52%)	15 (65%)	13 (57%)
Mean years of experience (SD)	10.3 (6.2)	19.5 (10.3)	18.6 (8.9)

*N = total number of participants per round; n = number of participants identifying with each expert category, recognizing experts identifying with multiple categories.*

### Data Collection and Analysis

#### Rounds 1 and 2

The opinions gathered from Round 1 resulted in 18, 28, and 26 grouped items for the reasons, characteristics, and uses of spotting, respectively. In Round 2, experts were asked to rate their agreement with the relevance of the grouped items from Round 1. There was 74–93% agreement on the relevance of 7 items of P1 (reasons for spotting), 71–100% agreement on 9 items of P2 (characteristics of successfully spotting), and 70–88% agreement on 10 items of P3 (uses of successfully spotting). The experts did not consensually disagree on the relevance of any items (i.e., 70% of experts rate an item 1 or 2, strongly disagree or disagree, on a 5-point Likert scale).

#### Round 3

The number of items resulting from Round 2 informed the BIBD design for the BWS in Round 3. Consequently, 7, 9, and 10 choice-sets of four items each were presented for the three prompts, respectively. The mean rankings (of the relevant items) can be seen in Table 2. Ultimately, weak consensus was found among the group. Low levels of consensus in the final rankings were observed for all three prompts (P1: W = 0.09, *p* = 0.06; P2: W = 0.08, *p* = 0.06; and P3: W = 0.13, *p* = <0.01). A Friedman’s Test indicated that the items regarding uses of successfully spotting were rated differently in P3, χ^2^ (9, *N* = 23) = 26.80, *p* < 0.01, whereas items referring to reasons and characteristics of successfully spotting in P1 and P2 just failed to reach significance [P1: χ^2^ (6, *N* = 23) = 12.02, *p* = 0.06; P2: χ^2^ (8, *N* = 23) = 15.24, *p* = 0.06].

**TABLE 2:** Group mean rankings of relevant items and top-half rankings of dance scientists (DS) and practitioners (P) subgroupings.

Prompt	Group mean rankings	DS	P
(P1) *Why do dancers spot?*	1. To orient themselves in space.	1	3
	2. To avoid or reduce dizziness.	2	
	3. To maintain spatial clarity.	3	
	4. To reference a desired end-point.		1
	5. To give rhythm to the turns.		2
	6. To focus while turning.		
	7. To maintain frontal awareness.		
(P2) *Successfully spotting is characterized by:*	1. Remaining oriented in the space during and after the turn.	1	4
	2. Having a clear spotting point.	3	2
	3. Precisely timing the head movement in the turn.	4	
	4. Moving the head and neck freely.		3
	5. Isolating the head as the last body part to rotate, and then whipping the head around.	2	
	6. Keeping a clear rhythm in the head movement.		1
	7. Having a clear visual focus.		
	8. Fixating a point.		
	9. Eliminating or reducing dizziness.		
(P3) *Successfully spotting is useful for:*	1. Performing multiple turns.	3	1
	2. Maintaining orientation in space.	1	3
	3. Providing a sense of direction.	2	5
	4. Turning with precision.		2
	5. Defining beginning and ending facings.	4	
	6. Keeping rhythm.		4
	7. Avoiding or reducing dizziness.	5	
	8. Maintaining control.		
	9. Maintaining balance.		
	10. Enhancing focus.		

The experts in Round 3 were further split into two groups of dance scientists (*n* = 12) – individuals holding a higher education degree in Dance Science or Human Movement Science – and practitioners (*n* = 11) – both ballet teachers and professional ballet dancers without dance-scientific experience and solely practical expertise. Top-half rankings for each subgroup (e.g., the items ranked 1–3 of the seven items from P1) are additionally identified in Table 2. Though higher than the group consensus level, dance scientists’ consensus on the three prompts was low (P1: W = 0.23, *p* = 0.01; P2: W = 0.10, *p* = 0.29; and P3: W = 0.15, *p* = 0.06). A Friedman’s Test indicated that items of P1 (reasons for spotting) were rated differently, χ^2^ (6, *N* = 12) = 16.78, *p* = 0.01, yet those of P2 and P3 (characteristics and uses of successfully spotting) failed to reach significance [P2: χ^2^ (8, *N* = 12) = 9.67, *p* = 0.29; P3: χ^2^ (9, *N* = 12) = 16.40, *p* = 0.06]. The practitioner subgrouping presented the greatest range of consensus levels (albeit low) over the three prompts (P1: W = 0.04, *p* = 0.84; P2: W = 0.15, *p* = 0.12; and P3: W = 0.21, *p* = 0.01). While the Friedman’s Tests for reasons and characteristics of successfully spotting (P1 and P2) were insignificant [P1: χ^2^ (6, *N* = 11) = 2.79, *p* = 0.84; P2: χ^2^ (8, *N* = 11) = 12.72, *p* = 0.12], items regarding the uses of successfully spotting (P3) were found to be rated differently by practitioners, χ^2^ (9, *N* = 11) = 21.15, *p* = 0.01.

Nevertheless, when further examining the relevant items in Table 2, notable topics in each prompt can be identified. The reasoning for spotting included: orientation (Table 2, P1: items 1, 3, 4, and 7), reduction of dizziness (P1: item 2), and contributions to rhythm (P1: item 5). Successfully spotting was further characterized by: a freely isolated head movement (Table 2, P2: items 4 and 5), a rhythmically timed movement (P2: items 3 and 6), gaze specificity (P2: items 2, 7, and 8), and functional characteristics (P2: items 1 and 9). Finally, successfully spotting was deemed useful for: multiple rotations (Table 2, P3: item 1), orientation (P3: items 2, 3, and 5), rhythm (P3: item 6), reducing dizziness (P3: item 7), and maintaining balance and control (P3: items 8 and 9). The results will be further discussed in terms of these topics.

### Additional Expert Contributions

In addition to the items agreed to be relevant, it is worthwhile to mention some items that did not reach such distinction. As a reason (P1) and use (P3) of successfully spotting, some items claimed that spotting generates force and momentum in the turn. Though biomechanically improbable, the item still received 44 and 48% agreement in this expert group for P1 and P3, respectively. Other notable items that did not reach consensual levels of agreement in response to why dancers spot (P1) included: “To maintain balance” (59% agreement), “Because it is traditionally taught” (59% agreement), and “To present an appearance of aesthetic ease” (22% agreement). In response to P2, only 63% of experts agreed that successfully spotting was characterized by “Stabilizing the head and gaze for as long as possible.” Regarding the use of successfully spotting (P3), the items “Limiting vestibular impact” and “Staying in the same place” received 63 and 44% agreement, respectively.

Notable comments were also contributed at the end of the surveys. Three experts commented that the most important characteristics of spotting can be turn- and/or individual-specific. Additionally, two experts commented in Round 3 that somewhat vague items and items relating to similar topics (though different in wording) were difficult to differentiate in ranking.

## Discussion

The goal of the present study was to evaluate consensus on the reasons, characteristics, and uses of spotting in dance rotations with a ranking-type Delphi method survey. As items were ranked significantly differently in only few cases (three of nine analyzed questions and groupings) and consensus on rankings were low in all cases, the items deemed relevant will thus be discussed together by topic.

### Prompt 1: Why Do Dancers Spot?

The first prompt aimed to elicit unrestricted answers on the reason for spotting. Items relating to aspects of orientation were most predominantly represented. While the great emphasis on orientation is perhaps novel to spotting, the role of head movements in orientation during locomotion has been widely examined. In a variety of studies examining changing directions while walking, anticipatory head movements have been observed to precede the rotation of the body, such that the head arrives first at the intended orientation (Grasso et al., 1998; Imai et al., 2001; Hollands et al., 2002). Hollands et al. (2002) point out that the head houses the vestibular and visual sensory systems that provide crucial information for constructing one’s position in space. Therefore, proactively realigning the head in a desired direction establishes a head-centered frame of reference for the central nervous system, which can in turn be used to coordinate the reorientation of the rest of the body. The head is thus seen to play a crucial role in steering the body (Imai et al., 2001). Consequently, it could be suggested that a preceding, isolated head movement is also relevant to orienting full revolutions. During dancers’ rotations, the head orientation of spotting is generally directed toward the intended final orientation. After sustaining the prior fixation, the head whips around to arrive before the body and steers the dancer toward this final orientation. Further research would be needed to corroborate similarities in head-body coordination between partial and full turns.

Other reasons presented for spotting included reduction of dizziness and contributions to turn rhythm. While spotting’s influence on the reduction of perceived dizziness aligns with previously suggested and empirically confirmed findings in the dancer population, purposes for rhythm introduce alternate reasons for spotting. The openness of the prompt allowed for reasons of aesthetical appearance or historical accumulation of technique. However, such topics failed to reach consensual levels of agreement in Round 2. Ultimately, this expert group indicated that spotting is performed for predominantly functional reasons – orientation, reduction of dizziness, and rhythm – rather than aesthetical or historical reasons.

### Prompt 2: Characteristics of Successfully Spotting

The second prompt aimed to gather movement components that characterize the successful performance of spotting. Similar to the focus on sequential coordination of the head in walking turns, the timing and rhythm of such a head movement was found to be critical to spotting; as identified by McMillan (1972) that skilled dancers’ heads arrive to their spot earlier in the turn. Additionally, the free movement of the head in isolation of the body’s rotation was identified as an important feature. This is in line with the findings of Lin et al. (2014) that experienced dancers have greater dissociation of the head and shoulders during rotation, whereas novice dancers performed their turns more en bloc with the head and shoulders rotating together. Lin et al. (2014) support this finding with the explanation that a space-referenced head coordination allows for greater stabilization of the head, and thus enhanced processing of visual information. However, it is interesting to note that an item with such affect – characterizing spotting by “Stabilizing the head and gaze for as long as possible” – just failed to reach agreement as a relevant item to this group of experts.

To speculate why head isolation might be more important than head stabilization, one may reference the fundamental coordination and range of motion in the head and torso. On one end of the spectrum, an en bloc coordination with no apparent head isolation can be disadvantageous, as the independent and countered rotations (in all planes) of the head and the torso work together to orient and stabilize the head in space (Imai et al., 2001). Furthermore, immobilizing the head causes changes in the timing and rotation magnitude of the trunk coordination in turning steps (Hollands et al., 2001). On the other end of the spectrum, if one were to truly stabilize the head for as long as possible during full revolutions, this would require maximal rotation, or yaw, of the head. However, it is known from normative range of motion values of the head and neck that at maximal rotation, the tilt, or roll, of the head also slightly increases (Schneider et al., 1975). Recalling the advice of Laws (1978), it is important for the head to stay on the axis of rotation with a smaller moment of inertia to reduce the angular momentum absorbed from the turn when spotting. Therefore, the maximal displacement of the head for an elongated stabilization may in fact be inefficient for full revolutions, increasing the inertia of the head for a more disruptive spotting technique. From these conjectures, it can be posed that there is an optimal amount of isolation between the head and torso during spotting: enough to allow the dancer to momentarily stabilize the head in space, though within the bounds where the head movement has minimized effects on turning dynamics.

Moreover, the experts were in line with the findings of the visual dependence of spotting (Cicchella and Caminiti, 2015), claiming the importance of fixating a spotting point in clear focus. This emphasis on visual information is further supported by reports that dancers are more dependent on visual information for balance regulation than other athletes, such as gymnasts and judoists (Golomer et al., 1997; Perrin et al., 2002). In terms of attentional focus in pirouette performance, two studies examined the use of instructions that emphasized maintaining a visual reference point. Da Silva et al. (2017) found that children with a visual instruction performed superiorly to children who were instructed to keep their head stable, in line with the current finding that head stabilization is perhaps a less important factor. However, with the inclusion of a control group, the study of Denardi and Corréa (2013) observed no advantage of instructions focusing on the head and vision. Therefore, though vision has clearly been identified as an important factor to dancers, the beneficial aspects of vision during rotation – particularly in terms of learning – have yet to be identified.

While it has been found that the presence of a visual target generally improves postural stability in quiet stance (Schärli et al., 2012), the influence of a visual target on head-torso coordination has been additionally examined. Solomon et al. (2006) noted that in step turns, participants used a more en bloc strategy when turning toward a remembered target than toward a clear visual target. This finding highlights the important role of visual targets for isolated head coordinations. While head isolation and timing as well as the role of visual fixations have been examined in simple balance and turning tasks, future movement-based research is needed to describe these three aspects in full-body revolutions.

Although movement descriptions were expected from P2 (Successfully spotting is characterized by:), some items rather related the underlying functions of movement components, characterizing successfully spotting by remaining oriented or perceiving reduced dizziness. The contribution of these items highlights the preferred language of experts for discussing and describing spotting – that is, with a functional approach. Action-oriented functional movement analysis (FMA) promotes the understanding of movements as a means to solving a given task (Hossner et al., 2015). With FMA, each movement characteristic is tied to functional substantiations. For an example from high jump, if the goal of a high jumper is to reach the greatest height possible, they jump backwards in an arched position (movement characteristic) in order to place their center of mass high above the bar (functional substantiation; Schärli, 2017). From such a perspective, it is understandable that one may suggest a successful high jump to be characterized by the completion of a task goal – a high center of mass over the bar – or in a dance context, a successful spot to be characterized by remaining oriented. Therefore, while one might expect a prompt on the characteristics of spotting to result in movement components, or modalities for accomplishing task goals, the experts’ responses highlight the group’s focus on functionality and further substantiate spotting as a purposeful action.

### Prompt 3: Uses of Successfully Spotting

Thus, drawing on functionality, the third prompt aimed to elicit the potential uses of successfully spotting. In line with the groups aptitude for functional discussion, P3 resulted in the highest level of consensus (albeit low) and was the only prompt in which the whole group rankings were significantly differentiated. While the research to date analyzing spotting in dance-specific rotations has mainly focused on single pirouettes (i.e., McMillan, 1972; Lin et al., 2014; Cicchella and Caminiti, 2015), the experts determined that spotting is most useful for performing multiple rotations. The newly presented topics of orientation and rhythm appeared once again, alongside the long-claimed uses of reducing dizziness and maintaining balance and control. Interestingly, while maintaining balance was agreed to be a utility of successfully spotting, such an item failed to reach consensual levels of agreement as a motivating reason for why dancers spot (P1). Therefore, it could be suggested that while spotting can be useful for aiding balance, perhaps balance is not the primary reasoning for spotting.

Notably, the lasting stigma that spotting adds force or momentum to turns resulted from Round 1 and starkly split the group in Round 2. In line with the biomechanical analysis of turns, almost half of the experts disagreed with the claim. However, a comparable portion of experts agreed, perhaps regarding the anecdotal role of spotting to help the dancer “get around” for the final rotation. To explain such a phenomenon, empirical analysis is needed to uncover the influence of spotting during rotation.

Beyond group rankings, top-half rankings of dance scientists and practitioners revealed unique outlooks. Particularly, items relating to rhythm in all three prompts were consistently in the top-half rankings of the practitioners, whereas consistently in the bottom-half rankings of the dance scientists. This difference perhaps elucidates the lack of empirical focus on the rhythmical aspects of spotting and further strengthens the need for research on this practically important topic.

### Limitations

The principles for reduction of free responses from Round 1 aimed to maintain the language of the experts as closely as possible, resulting in a large number of items; including items similar in topic, presenting a function when a movement characteristic was expected, or vague in description. The retention of such items could have increased the challenge of ranking relative importance in Round 3, and ultimately influenced levels of consensus and differentiation. In order to prevent similar issues, an additional round for validating items could be included. Before rating agreement in Round 2, the grouped items from Round 1 would be sent back to the experts for review (Schmidt et al., 2001; Kobus and Westner, 2016). This optional stage was removed in the present design to reduce the number of rounds that may have increased chances of participant dropout. However, such a stage would have been valuable, as participant comments insinuated difficulty in comparing the presented terms. Furthermore, a 4- instead of a 5-point Likert scale would have strengthened conclusions of agreement and disagreement by motivating experts to take a stance on each item (Villiers et al., 2005). In this way, the relevant items would be more decisively identified in Round 2.

Additionally, limitations in the formation of the prompts should be addressed. While a pilot study was first conducted to refine prompts and ensure clarity in the questions, further improvements can nevertheless be recognized. The first prompt (P1: Why do dancers spot?) posed an open and neutral question regarding the reasons for spotting, a movement within an embodied artform. Ultimately, the expert group proposed predominantly *functional* reasons. While this finding further confirms spotting as a functional movement, it may have also introduced some confusion in the similarity between items of P1 (reasons for spotting) and P3 (uses of successfully spotting). Similarly, a few functional responses arose in P2 (characteristics of successfully spotting), which can be attributed to the suggested affinity of this expert group to functional language or to a misunderstanding of movement *characteristics*. Moreover, the generalization of the prompts referring to “successfully spotting” sought to gather key uses and characteristics of the technique in rotations. Identifying a particular style or turn in the prompt may have increased consensus, as some experts commented on the specificity of the technique to the turn performed. However, the broad aim allowed for unbiased guiding factors to be brought forth, such as the notably important use of successfully spotting for “Multiple rotations,” whereas current spotting research has only examined single rotations.

Finally, even though the number of rounds was kept to a minimum of three, 7 of 30 experts dropped out throughout the survey process. While central to the Delphi methodology, the process of multiple feedback rounds increases the potential of dropouts (Hsu and Sandford, 2007). A larger expert panel was selected to encompass the multiple expertise categories; however, it proved difficult to manage and should be considered that a slightly different group of experts partook in each stage of consensus building. Moreover, the diversity of this larger expert group may have attributed to the low levels of consensus.

## Conclusion

While experts in the present study reached only low levels of consensus and differentiation, the topics generated provide valuable insights on the characteristics and uses of successfully spotting in dance rotations. Considering why dancers spot, experts added reasons of orientation and rhythm – which have been previously neglected in the discussion of spotting – to the long-held notion of reduction of dizziness. A successful spotting technique was described by movement characteristics of head isolation and timing as well as gaze specificity. Experts additionally contributed functional characteristics, which emphasize the utility of spotting during rotation. Successfully spotting was deemed most useful for performing multiple turns, together with the previously suggested uses of maintaining balance and reducing dizziness as well as newly proposed uses for orientation and keeping rhythm. The prevalence of rhythm throughout the three prompts resulted from practitioners’ preference for the topic, which was otherwise neglected by dance scientists. The Delphi method proved some weaknesses in item development and participant retention. Nevertheless, the resulting novel contributions to the discussion of spotting highlight the value of the Delphi methodology for gathering and integrating expert inputs. Moreover, the findings of this study – identifying key characteristics and functionalities of successfully spotting – serve as meaningful hypotheses for future movement-based research. Such studies are currently underway to help inform best practice.

## Data Availability Statement

The datasets generated for this study are available on request to the corresponding author.

## Ethics Statement

The studies involving human participants were reviewed and approved by the ethics committee of the local Faculty of Human Sciences at the University of Bern, Switzerland. The participants provided their written informed consent to participate in this study.

## Author Contributions

CH designed and conducted the study, performed the data analysis, and wrote the original manuscript. AS supervised the study and contributed to the design, data analysis, and revisions of the manuscript. Both authors contributed to the article and approved the submitted version.

## Conflict of Interest

The authors declare that the research was conducted in the absence of any commercial or financial relationships that could be construed as a potential conflict of interest.

## References

[B1] Cicchella A. Caminiti C. (2015). Effect of different spotting heights on ballet pirouette performance. *Acta Kinesiol. Univ. Tartuensis* 21 19–30. 10.12697/akut.2015.21.03

[B2] Da Silva M. T. Lessa H. T. Chiviacowsky S. (2017). External focus of attention enhances children’s learning of a classical ballet pirouette. *J. Dance Med. Sci.* 21 179–184. 10.12678/1089-313X.21.4.179 29166988

[B3] Denardi R. A. Corréa U. C. (2013). Effects of instructional focus on learning a classical ballet movement, the pirouette. *J. Dance Med. Sci.* 17 18–23. 10.12678/1089-313x.17.1.18 23498353

[B4] Diamond I. R. Grant R. C. Feldman B. M. Pencharz P. B. Ling S. C. Moore A. M. (2014). Defining consensus: a systematic review recommends methodologic criteria for reporting of Delphi studies. *J. Clin. Epidemiol.* 67 401–409. 10.1016/j.jclinepi.2013.12.002 24581294

[B5] Golomer E. Dupui P. Monod H. (1997). The effects of maturation on self-induced dynamic body sway frequencies of girls performing acrobatics or classical dance. *Eur. J. Appl. Physiol. Occup. Physiol.* 76 140–144. 10.1007/s004210050226 9272772

[B6] Grasso R. Prevost P. Ivanenko Y. P. Berthoz A. (1998). Eye-head coordination for the steering of locomotion in humans: an anticipatory synergy. *Neurosci. Lett.* 253 115–118. 10.1016/S0304-3940(98)00625-9 9774163

[B7] Hammond S. N. Hammond P. E. H. (1999). Technique and autonomy in the development of art: a case study in ballet. *Dance Res. J.* 21 15–24. 10.2307/1478627

[B8] Hollands M. A. Patla A. E. Vickers J. N. (2002). “Look where you’re going!”: gaze behaviour associated with maintaining and changing the direction of locomotion. *Exp. Brain Res.* 143 221–230. 10.1007/s00221-001-0983-7 11880898

[B9] Hollands M. A. Sorensen K. L. Patla A. E. (2001). Effects of head immobilization on the coordination and control of head and body reorientation and translation during steering. *Exp. Brain Res.* 140 223–233. 10.1007/s002210100811 11521154

[B10] Hopper D. M. Grisbrook T. L. Newnham P. J. Edwards D. J. (2014). The effects of vestibular stimulation and fatigue on postural control in classical ballet dancers. *J. Dance Med. Sci.* 18 67–73. 10.12678/1089-313X.18.2.67 24844423

[B11] Hossner E. J. Schiebl F. Göhner U. (2015). A functional approach to movement analysis and error identification in sports and physical education. *Front. Psychol.* 6:1339. 10.3389/fpsyg.2015.01339 26441717 PMC4564696

[B12] Hsu C. C. Sandford B. A. (2007). The Delphi technique: making sense of consensus. *Pract. Assess. Res. Eval.* 12 1–8.

[B13] Imai T. Moore S. T. Raphan T. Cohen B. (2001). Interaction of the body, head, and eyes during walking and turning. *Exp. Brain Res.* 136 1–18. 10.1007/s002210000533 11204402

[B14] Kim J. Wilson M. A. Singhal K. Gamblin S. Suh C. Y. Kwon Y. H. (2014). Generation of vertical angular momentum in single, double, and triple-turn pirouette en dehors in ballet. *Sports Biomech.* 13 215–229. 10.1080/14763141.2014.933580 25325768

[B15] Kim K. S. Kim Y. H. Shin S. H. Choi J. S. Lee S. Jang T. Y. (2013). Post-rotatory visual fixation and angular velocity-specific vestibular habituation is useful in improving post-rotatory vertigo. *J. Int. Adv. Otol.* 9 175–179.

[B16] Kirker B. Tenenbaum G. Mattson J. (2000). An investigation of the dynamics of aggression: direct observations in ice hockey and basketball. *Res. Q. Exerc. Sport* 71 373–386. 10.1080/02701367.2000.10608920 11125535

[B17] Kobus J. Westner M. (2016). “Ranking type Delphi studies in IS research: step-by-step guide and analytical extension,” in *Proceedings of the 9th IADIS International Conference Information Systems*, eds Nunes M. B. Isaías P. Powel P. (Vilamoura: IADIS), 28–38.

[B18] Kschessinka M. (1997). *Dancing in Petersburg: The Memoirs of Kschessinska / H. S. H. the Princess Romanovsky-Krassinsky.* New York, NY: Da Capo Press.

[B19] Laws K. (1978). An analysis of turns in dance. *Dance Res. J.* 11 12–19. 10.2307/1477841

[B20] Laws K. (1986). The mechanics of the fouetté turn. *Kinesiol. Dance* 28 22–24.

[B21] Lee J. A. Soutar G. Louviere J. (2008). The best-worst scaling approach: an alternative to Schwartz’s values survey. *J. Pers. Assess.* 90 335–347. 10.1080/00223890802107925 18584442

[B22] Lerch K. Khastoo A. Rowley K. M. Kulig K. (2017). “Exploring the Saut de chat leap: a Delphi method study,” *Poster Session Presentation at the Meeting of the International Association of Dance Medicine and Science*, Houston, TX.

[B23] Lin C. W. Chen S. J. Su F. C. Wu H. W. Lin C. F. (2014). Differences of ballet turns (pirouette) performance between experienced and novice ballet dancers. *Res. Q. Exerc. Sport* 85 330–340. 10.1080/02701367.2014.930088 25141086

[B24] Louviere J. Lings I. Islam T. Gudergan S. Flynn T. (2013). An introduction to the application of (case 1) best–worst scaling in marketing research. *Int. J. Res. Market.* 30 292–303. 10.1016/j.ijresmar.2012.10.002

[B25] Mayring P. (2000). Qualitative content analysis. *For. Qual. Soc. Res.* 1:20.

[B26] McMillan M. H. (1972). *A Cinematographic Analysis of Characteristic Likeness and Differences Between Skilled, Semi- Skilled, and Non-Skilled Performances of Pirouettes.* Master’s thesis. Denton, TX: Texas Women’s University.

[B27] Murry J. W. (1995). Delphi: a versatile methodology for conducting qualitative research. *Rev. High. Educ.* 18 423–426. 10.1353/rhe.1995.0008

[B28] Nigmatullina Y. Hellyer P. J. Nachev P. Sharp D. J. Seemungal B. M. (2015). The neuroanatomical correlates of training-related perceptuo-reflex uncoupling in dancers. *Cereb. Cortex* 25 554–562. 10.1093/cercor/bht266 24072889 PMC4380084

[B29] Obrist D. (2011). *Fluid Mechanics of the Inner Ear.* Dissertation. Zurich, CH: ETH Zurich.

[B30] Osterhammel P. Terkildsen K. Zilstorff K. (1968). Vestibular habituation in ballet dancers. *Acta Oto-laryngol.* 66 221–228. 10.3109/00016486809126289 5304145

[B31] Paré G. Cameron A. F. Poba-Nzaou P. Templier M. (2013). A systematic assessment of rigor in information systems ranking-type Delphi studies. *Inform. Manag.* 50 207–217. 10.1016/j.im.2013.03.003

[B32] Perrin P. Deviterne D. Hugel F. Perrot C. (2002). Judo, better than dance, develops sensorimotor adaptabilities involved in balance control. *Gait Post.* 15 187–194. 10.1016/S0966-6362(01)00149-7 11869913

[B33] Schärli A. (2017). “Functional movement analysis in dance,” in *Handbook of Human Motion*, Vol. 13 eds Müller B. Wolf S. I. Brueggemann G.-P. Deng Z. McIntosh A. Miller F. (Heidelberg: Springer), 1–15. 10.1007/978-3-319-30808-1_112-1

[B34] Schärli A. van de Langenberg R. Murer K. Müller R. M. (2012). The influence of gaze behaviour on postural control from early childhood into adulthood. *Gait Post.* 36 78–84. 10.1016/j.gaitpost.2012.01.008 22326471

[B35] Schmidt R. Lyytinen K. Keil M. Cule P. (2001). Identifying software project risks: an international Delphi study. *J. Manag. Inform. Syst.* 17 5–36. 10.1080/07421222.2001.11045662

[B36] Schneider L. W. Foust D. R. Bowman B. M. Snyder R. G. Chaffin D. B. Abdelnour T. A. (1975). Biomechanical properties of the human neck in lateral flexion. *SAE Trans.* 84 3212–3224.

[B37] Solomon D. Kumar V. Jenkins R. A. Jewell J. (2006). Head control strategies during whole-body turns. *Exp. Brain Res.* 173 475–486. 10.1007/s00221-006-0393-y 16506002

[B38] Teramoto K. Sakata E. Ohtsu K. (1994). Use of the visual suppression test using post-rotatory nystagmus to determine skill in ballet dancers. *Eur. Arch. Oto-Rhino-Laryngol.* 251 218–223. 10.1007/BF00628427 7917255

[B39] Tokita T. Watanbe T. Fukuda T. (1972). Telemetering of eye and head movements in ballet rotation. *Agressologie* 13 21–27. 5078826

[B40] Tschiassny K. (1958). Perrotary nystagmus in the ballet dancer, pigeon, and blind person. *Ann. Otol. Rhinol. Laryngol.* 66 641–648. 10.1177/000348945706600303 13488339

[B41] Villiers M. R. de, Villiers P. T. de Kent A. P. (2005). The Delphi technique in health sciences education research. *Med. Teach.* 27 639–643. 16332558 10.1080/13611260500069947

